# A Standardized Protocol for Stereotaxic Intrahippocampal Administration of Kainic Acid Combined with Electroencephalographic Seizure Monitoring in Mice

**DOI:** 10.3389/fnins.2017.00160

**Published:** 2017-03-29

**Authors:** Pascal Bielefeld, Amanda Sierra, Juan M. Encinas, Mirjana Maletic-Savatic, Anne Anderson, Carlos P. Fitzsimons

**Affiliations:** ^1^Neuroscience Program, Faculty of Sciences, Swammerdam Institute for Life Sciences, University of AmsterdamAmsterdam, Netherlands; ^2^Achucarro Basque Center for NeuroscienceZamudio, Spain; ^3^Ikerbasque FoundationBilbao, Spain; ^4^University of the Basque Country (UPV/EHU)Leioa, Spain; ^5^Baylor College of Medicine, The Jan and Dan Duncan Neurological Research Institute at Texas Children's HospitalHouston, TX, USA

**Keywords:** kainic acid, adult hippocampal neurogenesis, EEG, intrahippocampal administration, epilepsy, temporal lobe

## Abstract

Lack of scientific reproducibility is a growing concern and weak experimental practices may contribute to irreproducibility. Here, we describe an optimized and versatile protocol for stereotaxic intrahippocampal administration of Kainic Acid (KA) in mice with a C57Bl6 background. In this protocol, KA administration is combined with *in vivo* recording of neuronal activity with wired and wireless setups. Following our protocol, KA administration results in a robust dose-dependent induction of low-level epileptiform activity or Status Epilepticus (SE) and induces previously characterized hallmarks of seizure-associated pathology. The procedure consists of three main steps: Craniotomy, stereotaxic administration of KA, and placement of recording electrodes in intrahippocampal, and subdural locations. This protocol offers extended possibilities compared to the systemic administration of KA, as it allows the researcher to accurately regulate the local dose of KA and resulting seizure activity, and permits the use and study of convulsive and non-convulsive KA doses, resulting in higher reproducibility and lower inter-individual variability and mortality rates. Caution should be taken when translating this procedure to different strains of mice as inter-strain sensitivity to KA has been described before. The procedure can be performed in ~1 h by a trained researcher, while intrahippocampal administration of KA without placing recording electrodes can be done in 25 min, and can be easily adapted to the titrated intrahippocampal administration of other drugs.

## Introduction

Many available animal models of Mesial Temporal lobe epilepsy (mTLE), and in particular systemic KA administration, are induced by acute treatments that often are associated with high mortality and experimental variability rates (Hellier et al., 1998; McLin and Steward, 2006; Kienzler-Norwood et al., 2017). Modeling mTLE using experimental animals comprises, in general, three well-defined stages: First, an initiating insult to the brain, such as febrile seizures, head trauma, stroke, or strong seizures leading to status epilepticus (SE). This initial insult triggers a cascade of events resulting in a second, latent period, during which molecular and structural changes take place, commonly known as epileptogenesis. Finally, a third period is characterized by the occurrence of spontaneous seizures, resulting in chronic epilepsy (Raedt et al., 2009).

Several protocols are extensively used in preclinical research to characterize epilepsy development, such as brain infarction, traumatic brain damage, febrile seizures, electrical kindling, or administration of chemoconvulsants such as the cholinomimetic pilocarpine and the systemic or intrahippocampal administration of the glutamate agonist kainic acid (KA) (Parent et al., 1997; Hellier et al., 1998; Bouilleret et al., 1999; Hartings et al., 2003; Kharatishvili et al., 2007; Dubé et al., 2010; Morales et al., 2014). Each model has been developed and characterized to have their own distinctive properties, including working mechanism and/or pharmacokinetics, and each one of them may model different aspects of epilepsy, providing clear advantageous alternatives. On the other hand it is, in some cases, hard to compare and combine data across models. Furthermore, several of the above-mentioned models induce varying mortality rates and are accompanied by severe animal welfare issues (Curia et al., 2008; Lévesque and Avoli, 2013; Lidster et al., 2016). Therefore, preclinical epilepsy research would benefit from more detailed and standardized protocols, with lower mortality rates and improved animal welfare, which could increase intra- and inter-laboratory reproducibility (Lidster et al., 2016; Kienzler-Norwood et al., 2017).

Here, we describe a detailed protocol for the intrahippocampal administration of KA in mice, a well-characterized preclinical epilepsy model that mimics major aspects of mTLE, in which seizures originate in the hippocampus and/or related limbic areas and which was first described by Bouilleret et al. (1999). We have adapted the protocol to optimize the delivery of KA to the dentate gyrus (DG), where local delivery of KA induces the expression of several parameters associated with seizure pathology, such as occurrence of spontaneous recurrent seizures, gliosis, granule cell dispersion, and strong alterations in resident neural stem cells, e.g., strong changes in gene expression, neural stem cell activation, and subsequent depletion of the hippocampal neural stem cell pool (Schouten et al., 2015; Sierra et al., 2015). Experiments using this protocol in combination with KA doses high enough to induce mTLE have demonstrated the generation of spontaneous seizures and interictal discharges that started 6 h after KA injection and persisted for up to 50 days (Sierra et al., 2015). Therefore, our protocol may be applicable as a model for chronic epilepsy studies associated with alterations in adult neurogenesis and epileptic seizures.

**Figure 1:**
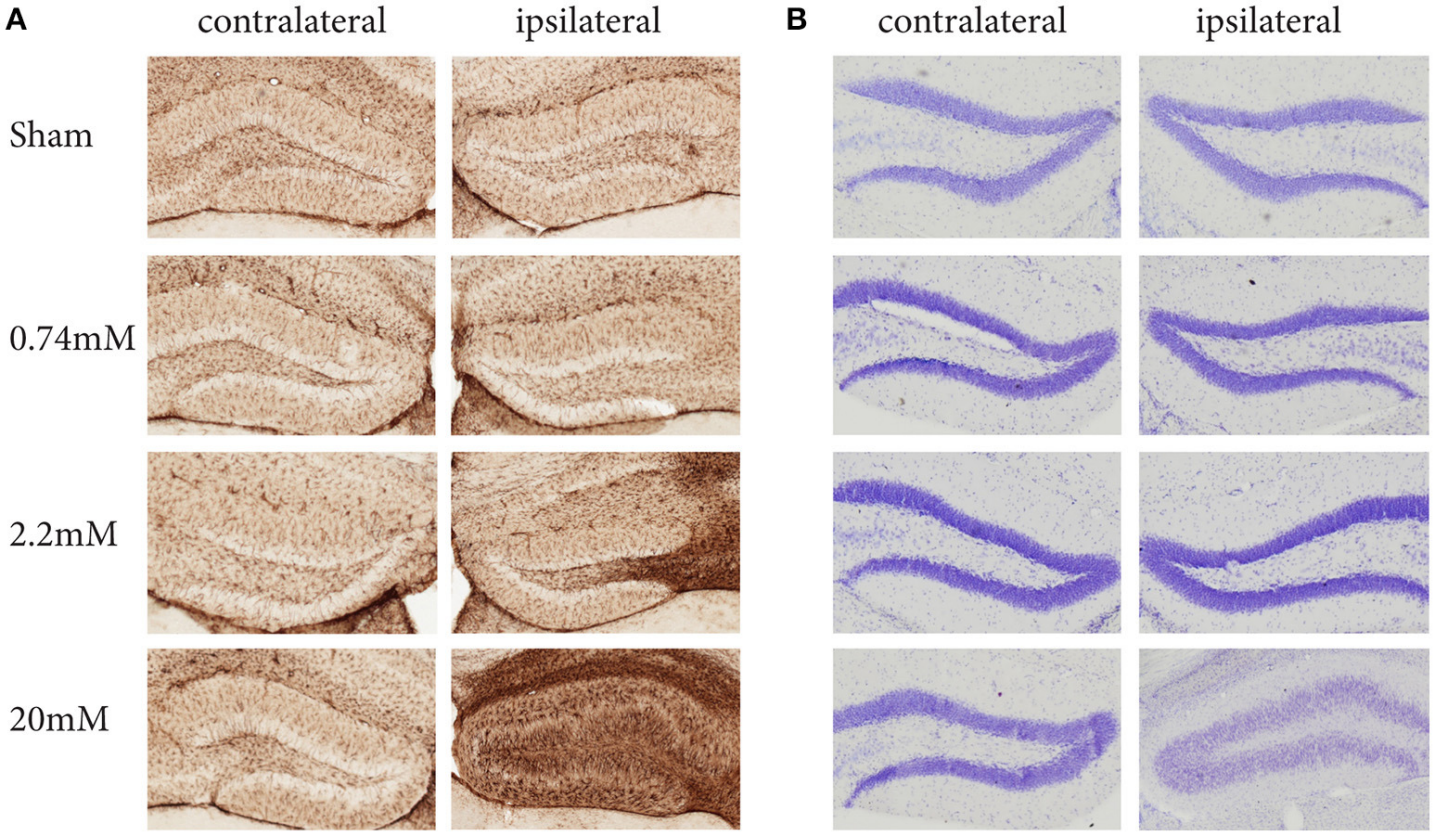
**Kainic Acid dose-dependent effects on gliosis (A)** and granule cell dispersion **(B)**, 28 days post KA administration. Immunohistochemistry against GFAP reveals clear dose-dependent gliosis effects, becoming apparent at 2.2 mM KA, which coincides with the presence of epileptic seizures. A Nissl staining to assess granule cell dispersion only shows granule cell dispersion when administering 20 mM KA, while the seizure-inducing 2.2 mM shows no sign of dispersion. Interestingly, for both gliosis and granule dispersion, the effects are limited to the ipsilateral hemisphere.

The protocol we describe here has been carefully standardized and reproduced independently in three research centers. It describes the local administration of KA into the mouse hippocampus using stereotaxic injection and simultaneous recording of EEG activity. Depending on the KA dose used, this protocol induces different levels of epileptiform activity and hippocampal alterations, such as granule cell dispersion (GCD) and gliosis (Figure 1). Previous observations have demonstrated that the protocol results in local regulation of apoptosis, inflammation, chronic spontaneous seizures, as well as strong alterations in resident neural stem cells, substantially affecting their neurogenic potential. At the molecular level, these alterations are reflected in significant changes in gene expression (Schouten et al., 2015, 2016; Sierra et al., 2015).

Compared to other commonly used epilepsy models, in particular the systemic administration of KA, this model offers several advantages: (1) SE is initiated in the hippocampus, whereas systemic convulsants may affect many brain areas at the same time (including the cortex and limbic areas), complicating the interpretation of results. (2) In addition, by modifying the stereotaxic coordinates used for KA delivery, this protocol may be adapted to model epilepsy in other brain areas. (3) The protocol describes the use of three doses of KA, which give the possibility to induce neuronal hyperactivity at different intensities. (4) In comparison with the systemic administration of KA, the protocol allows for uni- or bi-lateral KA administration, sparing the contralateral hippocampus as undamaged tissue control in the short term (3 days post-injection, Figure 1). However, the pathological changes induced by KA may extend to the contralateral hippocampus at longer times post-injection. (5) The protocol is dose-optimized in mice and thereby it can be applied to numerous easily accessible transgenic mice lines of interest. Regarding this last point, we recommend caution to be applied while comparing different mouse strains, as KA-sensitivity has been reported to differ between them, at least when administered systemically (McLin and Steward, 2006; Schauwecker, 2014). Finally, this model has the disadvantage of potentially inducing local tissue damage around the needle track, which we minimize by the use of glass pulled microcapillaries, as originally described by Bouilleret et al. (1999), resulting in minimal neuronal damage and local inflammation-induced glial and neural stem cell reactivity (Sierra et al., 2015).

## Materials

- Kainic Acid Monohydrate (K0250, Sigma Aldrich)^#^
- Sterile Saline (Eurovet)
- Ethanol 70% (disinfectant)
- Mice (C57Bl6/J, Harlan, The Netherlands)
- Betadine (Premed healthcare B.V.)
- Anesthetics and Analgesics: Isoflurane, Lidocaine, Ketamine (Ketolar, Pfizer), xylazine (Sigma Aldrich), Buprenorphine (Buprecare, Animalcare Ltd)
- Lubricant eye ointment (CEVA Netherlands)
- Dental Cement (Simplex Rapid, Kemdent)
- Surgical PVDF sutures (PRONOVA, Ethicon)^*^
- Cotton swabs (Servoprax, Germany)
- Microlance injection needles, with bent tip (25G) (BD)
- Mineral Oil (M5904, Sigma Aldrich)

### Equipment for surgery and injections

- Isoflurane anesthesia induction box
- Surgical Tools, including small surgical scalpel, scissors, and forceps
- Watchmaker screwdriver set
- #5 Dumont forceps (Fine Science Tools, Foster City, CA, USA)
- 1.5 mm diameter Micro curette (Fine Science Tools)
- Electrical hair shaver
- Small animal digital stereotaxic apparatus (Kopf model 940 or Stoelting model 51730) equipped with a large probe holder (Stoelting 51633) and a cannula holder (Stoelting 51636)
- Heating pad
- Dissecting Microscope (Olympus SZ61)
- Borosilicate glass capillaries (I.D. 0.53 mm, O.D. 1.14 mm; 3-000-203-G/X, SuedLabor, Germany)
- Nanoject II Auto-Nanoliter injector (Drummond Scientific Company)
- Hand-held drill (Kopf model 1474-220) with matching drill bits (drillhead 0.6 mm/0.024 inch and 0.81 mm/0.032 inch)
- Micropipette puller (model P-87, Sutter Instruments)
- Syringes (1.0 mL) with 25 G needle

### Equipment for wireless recording

**Figure 2:**
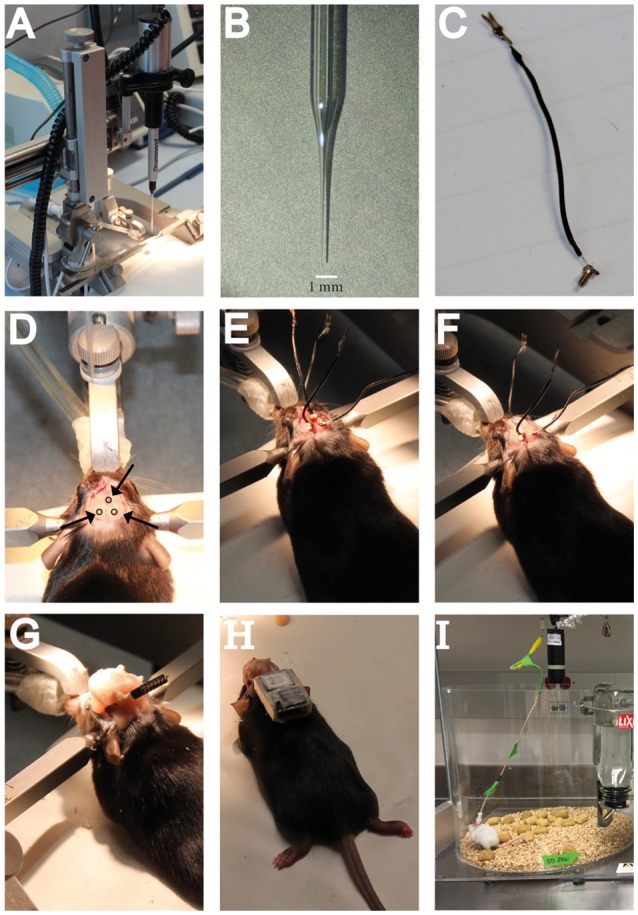
**Hardware needed for intrahippocampal administration of KA and a guideline including crucial steps in the surgery procedure for electrode implantation and EEG recordings**. **(A)** The stereotaxic setup in which the NanoJect is placed. **(B)** Close-up image of the injection capillary. **(C)** Example of a recording electrode as used for the subdural wireless EEG recordings. **(D)** Location of the burr holes in the skull as used for the wireless EEG electrode implantation. **(E)** Placement of the subdural recording electrodes in the burr holes. **(F)** Attaching the recording electrodes to the skull with dental cement. **(G)** Covering the recording electrodes in dental cement and attachment of the pin connector. **(H)** Example of a mouse carrying a wireless recording device. **(I)** Example of a mouse in the wired EEG recording setup.

- Wireless EEG recorders (Neurologger, TSE systems, Germany)
- Neurologger Read Out Station (TSE systems, Germany)
- Wired recording electrodes consisting of 0.8 mm shaft diameter gold plated screws, attached to 1.2 cm long electrode wire soldered to connector pins matching the Neurologger (TSE Systems, Germany) (Figure 2C).
- One wired reference/ground electrode that consists of two separate wires attached to connector pins soldered to one gold-plated screw.

### Equipment for wired recording

- Nicolet video-EEG system (NicView 5.71, CareFusion, San Diego, CA, USA) or Cereplex video- EEG system (Blackrock Microsystems, Salt Lake City, UT, USA)
  Six channel cable (363-441/6, PlasticsOne, Roanoke, VA, USA)
- 5 mm long (1 mm stripped) platinum iridium, Teflon-coated deep electrodes (E363/8; PlasticsOne). Teflon-coated electrodes can be purchased 5 mm long and later on cut at the desired length with the help of a ruler and a razor blade (2 mm for the hippocampi, 1 mm for the cortices). Do not forget to strip the last mm of teflon by cutting it away with a razor blade (without damaging the electrode) and pulling from the tip of the teflon with forceps.
- Plastic pedestal (MS363) and dust cap (363DC; Plastics One)
- Plastic pedestal holder (MH-636)
- Stainless steel mounting screws (00-96x1/16; Plastics One); 1.6 mm shaft length, 2.16 mm head diameter; 1.19 mm shaft diameter.

## Procedure

All animal procedures were performed following the European directive 2010/63/UE and NIH guidelines and were approved by the respective institutional Ethics Committees. Animal procedures described herein are registered and approved by the animal health and care committee, University of Amsterdam (protocol #296).

### Preparation of surgery area, equipment, and materials

Before starting the surgical procedure make sure to thoroughly disinfect the surgical table and any equipment that will be used. 70% ethanol suffices for surfaces, while tools preferably are sterilized by autoclaving.

1. Prepare the KA solution to be used for injection. This protocol uses three different doses of KA, each showing differential EEG activity. The highest concentrations used (20 mM) induces status epilepticus and chronic seizures, while the intermediate dose (2.22 mM; 1:9) induces mild non-convulsive behavioral status, and the lowest dose (0.74 mM, 1:27) only results in enhanced epileptiform activity (Schouten et al., 2015; Sierra et al., 2015).
2. Prepare KA stock solution by dissolving Kainic Acid Monohydrate in sterile Saline. Prepare working stocks (10 uL) and store at −20°C.
3. Pull the glass capillaries to create injection needles. Injection needles should be optimized in such a way that a very long and thin shaft is obtained to reduce tissue damage (Figure 2B). Fill one with mineral oil and attach it to the Nanoject injector. The mineral oil is non-compressible and allows the pressure of the injector to be evenly distributed, providing exact injection volumes in the nanoliter range.

#### Anesthesia and placement in the stereotaxic apparatus

We recommend using isoflurane anesthesia as this allows for a tight control of the anesthesia plane and the fastest recovery after surgery. Alternatively, if an isoflurane setup is not at your disposal it is also possible to use ketamine/xylazine (Sierra et al., 2015), although ketamine is a NMDA channel blocker and may potentially delay the initiation of seizures induced by KA. Whichever the choice, the same anesthesia procedure should be used for all animals within each experiment.

4. Weigh the animal before surgery to allow valid control of post-surgical weight loss.
5. Place the animal in the isoflurane induction box (5% isoflurane, 95% air, 250 CC/min) until the animal is fully anesthesized. The animal should no longer be responsive to nociceptive stimuli, such as pinching the toes with forceps. Alternatively, inject with ketamine/xylazine (10/1 mg/kg, intraperitoneal). As soon as the mouse is immobile, inject with buprenorphine (1 mg/kg, subcutaneous), as the combination of drugs leads to a deeper anesthesia. If the mouse is not fully anesthetized within 10 min, re-inject with ketamine alone (50–100 ul of 100 mg/ml) to avoid a xylazine overdose that would result in bradychardia and respiratory depression.
6. Proceed by shaving the fur of the skull, disinfect the skin with betadine and place the animal in the stereotact. Make sure the animal is placed horizontally and symmetrical to the earbars by pulling from the base of its tail lightly before screwing the earbars and nosebar tightly.

### Craniotomy

While performing the craniotomy and the subsequent steps of this protocol make sure to monitor the general condition of the animal. Make sure anesthesia is sufficient, but not too deep, to allow a quick recovery from anesthesia. During these steps, adjust the isoflurane percentage and/or flow when needed. As a general guideline, 2% isoflurane at a flow of 250 CC/min should be used. When using ketamine/xylazine it is usually not necessary to re-anesthetize the animal because once deep anesthesia has been reached it will last for 20–30min.

7. Start by making a midline incision from the frontal cranial bones to the back of the parietal cranial bones using a small blade scalpel. Push the skin aside so that the skull becomes visible. To improve analgesia and reduce animal discomfort, the local administration of lidocaine to the skull periost is recommended by animal welfare officers in The Netherlands. Discuss the use of local lidocaine with your local animal welfare officer. After 5 min, use small forceps and cotton swabs to remove the periost and clean the skull surface.
8. Place the Nanoject Injector with the injection capillary in the large probe holder of the stereotaxic fame (Figure 2A). Identify the skull sutures and both bregma and lambda. Make sure the head of the animal is leveled in the rostral-caudal direction by measuring the *Z* coordinates of both bregma and lambda and adjust the nosebar if needed. Follow the same procedure for the horizontal positioning of the animal by checking the *Z* coordinates at the midline and two corresponding locations on the left and right side of the midline. Horizontal positioning can only be adjusted, if needed, by repositioning the animal in the apparatus and readjusting the earbars.
9. When the animal is correctly positioned, proceed to identify the coordinates of choice on the skull surface. In our case, using P42 male C57BL/6 mice, we use the following coordinates to target the DG: Anteroposterior (AP) −2.0, mediolateral (ML)+1.5 mm, dorsoventral (DV) −2.0 mm (Schouten et al., 2015), or AP −1.7, ML +1.6, DV −1.9 (Sierra et al., 2015) (both based on the Paxinos and Franklin Brain Atlas). Using these coordinates KA infusion targets the suprapyramidal blade of the dorsal DG. No differences in the SE or epileptogenic activity of the hippocampus have been found when comparing both protocols (Schouten et al., 2015; Sierra et al., 2015). Besides the burr hole needed for KA infusion, also drill holes for the recording electrodes are necessary. For the wireless recording of the hippocampus, drill one burr hole contralateral to the KA infusion site (AP −2.0 mm, ML −1.5 mm), and another hole for the reference electrode (AP +0.1, ML +0.1) (Figure 2D).
  For the wired recordings of the cortex and hippocampus, drill the holes at AP −0.1 mm, ML +1.6 mm (left cortex), AP −0.1 mm, ML −1.6 mm (right cortex), and AP −1.8 mm, ML +1.6 mm (left hippocampus). Drill a hole for the reference electrode in the frontral cortex at AP +0.1 mm, ML +0.1 mm. Drill two additional holes for the mounting screws at approximately AP +3.0, ML +6.0 and AP −8.0, ML −0.5. It is important to place the mounting screws far away from where the electrode pedestal will be positioned, to avoid contact between the screws and the electrodes that may produce interference with the electric signals recorded from the brain.
10. Use the handheld drill to carefully drill holes in the skull at the coordinates identified previously. Be careful while applying pressure, as drilling through the bone will likely cause damage to the brain parenchyma. Stop when you are almost through the bone (blood vessels become clear). Use a bent injection needle to further open the burr hole, and remove any bone tissue with small forceps. Furthermore, make sure to open up the dura, as this will interfere with the smooth and straight insertion of the injection capillary.

### Intrahippocampal injection

11. Use the nanoject controller to fill the capillary with the KA solution. Before injecting the KA, make sure to set the right settings on the controller and push a few solution drops to drain any possible air bubble at the end of the capillary. We use the following nanoject settings: 4.6 nL/injection at slow speed. Gently lower the capillary and identify the *Z* coordinate for the brain surface. From there, lower the capillary to -2.0 mm. Using the Nanoject controller, inject 50 nL (11 × 4.6 nL, every 10 s). Given the small total injection volume, there is no need to wait with retracting the injection capillary, as no backflow will occur.

### Insertion of recording electrodes

From this point on, one has the option to implant electrodes of choice. In this protocol we describe two different approaches, both of which have been used in recent research publications. We describe first a subdural neocortical wireless recording technique, labeled A and previously used in Schouten et al. (2015) and an intrahippocampal and intracortical wired recording, labeled B and previously used in Sierra et al. (2015).

#### Subduralneocortical wireless recordings

11a. Using a watchmaker screwdriver, place the recording electrodes in the burr holes created before. Place them in such way that the tip of the electrode makes contact with the brain surface (Figure 2E). It is important that all electrodes are placed at the same depth, to ensure comparable subdural recordings without creating tissue damage. The exact depth and placement will depend on the electrodes used, so electrode placement should be optimized before the experiments. Gold-plated screws with a diameter comparable, or slightly bigger, than the head of the drill bit should be used, so that the screws fix themselves on the bone.
12a. Once all electrodes are in place, completely dry the skull surface and gently cover the electrodes with sufficient dental cement to strengthen their hold on the skull. It is important to let the cement dry completely before proceeding to the next step (Figure 2F).
13a. Connect the electrodes to the pin connector and gently wind up the electrode wire in such a way that they take up as little place as possible, and the pin connector is positioned horizontally and pointing backwards. The position of the pin connector is very important, as the Neurologger will be attached later, and weight distribution of the logger is essential to minimize animal discomfort and free movement. When the wire and pin connector are in position, fix the structure with dental cement (Figure 2G). This will not only strengthen the connection to the skull, but will also protect the electrodes from damage.

#### Intrahippocampal and intracortical wired recordings

11b. Screw the stainless steel mounting screws in their holes with the watchmaker screwdriver by holding them from their head with the Dummont forceps.
12b. Place the six electrodes in the electrode pedestal with the help of the Drummond forceps in their correct position. Use 2 mm long electrodes for the hippocampi, 1 mm electrodes for the cortices and the reference electrode, and a bent 5 mm electrode for the ground electrode that will be positioned over the cervical paraspinous area. Attach the plastic pedestal to its holder, and this piece to the cannula holder of the stereotaxic apparatus. Make sure the pedestal holder is correctly aligned to the vertical axis of the stereotaxic frame. Draw a vertical line over the midline of the pedestal holder to facilitate the visual alignment.
13b. Lower the pedestal holder so that the five deep electrodes are positioned right above their drill holes. Make sure the five are on their correct position pushing them lightly with the help of the Drummond forceps. Lower the pedestal to its final position contacting the skull. Apply dental cement with the micro curette around the pedestal and screws, creating a small mount covering the skull exposed right up to the skin borders. Make sure the dental cement does not cover the skin to prevent the pedestal to become detached over time. While creating the mount, keep the dental cement to be applied in a semiliquid state by adding more solvent as necessary. Thick dental cement will produce a crumbly mount, while liquid dental cement will be too slippery to be applied in the right position. Cover the pedestal with the plastic pedestal cap.
14a/b. Let the dental cement dry out completely before removing the animal from the stereotaxic apparatus. Keep the animal warm using an electric heat pad until it is awake and ambulant (5 min in A, 30 min–1 h in B).

### EEG recording and data collection

#### Wireless recording

15a. Attach the pin connector on the mouse skull to the Neurologger (Figure 2H). The wireless Neurologger provides 72 h of continuous recording at a sampling rate of 500 Hz. Once every 3 days the batteries have to be replaced. While replacing the batteries, collect data from the recorder using the Neurologger ReadOut station. Neurologger software provides plugins to convert data into Matlab compatible files for analysis.

#### Wired recordings

15b. Immobilize the mouse down to a flat surface by holding its back with the hand palm and both sides of the pedestal with the index and thumb. If necessary, a light anesthesia can be administered (2% isofluorane in an induction chamber for 30 s). Remove the pedestal cap and attach the pedestal to the six channel cable connected to the Nicolet or Cereplex recorders. The mouse can be recorded at different sampling rates in 4 h sessions in a cage without access to food or water to prevent the cable from tangling, for up to 50 days (Figure 2I).

### Potential pitfalls and troubleshooting

The protocol described here makes use of general stereotaxic surgery procedures and therefore most of its potential pitfalls have been described elsewhere (Cetin et al., 2007). However, several specific points have to be closely monitored in order to perform the procedure correctly.

**Figure 3:**
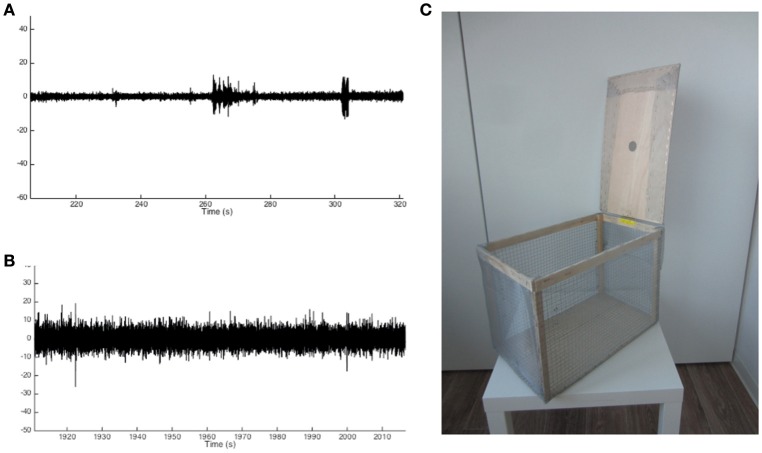
**Example traces of noise-free and noisy EEG recordings and Faraday cage design**. **(A)** Example trace of a noise-free recording showing small deviations from baseline activity. **(B)** Example trace of a noisy recording showing significant deviations from baseline activity to an extent that may overlap with changes from baseline in the noise-free recording. **(C)** Example of a Faraday cage used to prevent noisy EEG recordings.

1. The choice of anesthetics is crucial for interpreting the obtained results. As mentioned above the use of Isoflurane anesthesia is recommended, as isoflurane does not affect ion-channel function, and therefore does not interfere with seizure induction. However, an isoflurane setup will not always be available, which may influence the choice of anesthetics. As we have shown before, Ketamine/Xylazine anesthesia provides a widely used alternative (Sierra et al., 2015; Abiega et al., 2016). However, potential differences in status epilepticus delay and/or intensity may arise when comparing isofluorane and ketamine anesthetized mice.
2. The placement of the animal in the stereotaxic apparatus is crucial for proper localization of the injection capillary. If the animal is not correctly placed, the injection coordinates will not correspond to the area of interest. It is crucial to always check the rostral-caudal and medial-lateral alignment using the dorsal-ventral stereotaxic coordinates.
3. Though the anesthesia procedures described in this protocol have been thoroughly standardized, animal well-being must always be monitored very closely. Anesthetics dosages described here provide a general guideline, though differences in inter-animal sensitivity to anesthesia occur. Therefore, always monitor breathing and heart rate of the animal throughout the procedure, and adjust anesthetics if necessary.
4. Since the injection volume is very small and the injection capillary diameter is very narrow it is important to closely monitor the tip of the capillary prior to and during the procedure. First, because of the small diameter of the glass pipette, capillary action might result in displacement of the KA solution in the capillary tip by blood or other liquids encountered on the brain surface. This can be easily monitored under the microscope while lowering the injection capillary onto the brain surface. If capillary action occurs always retract the capillary and reload the KA solution. Secondly, the glass pipette can easily clog because of its small diameter. Before inserting the capillary always check whether clogging occurred by performing a test-injection outside of the skull.
5. Placement of the electrodes is crucial for correct electrophysiological recordings. In the wired approach described here electrodes are placed in the brain, and therefore the same pitfalls apply as for the KA injection itself. For the wireless, neocortical approach described, placement of the electrodes has to be optimized, as the electrode screws have to be placed touching the brain surface. Since these procedures cannot be visually monitored, electrode placement has to be checked post-mortem. In our wireless setup and using the gold-plated screws described here, two full turns with a screwdriver delivered optimal results. When using other screws, mice of other age, or other rodent species in which the skull thickness might differ, this procedure must be re-optimized.
6. Because the recording electrodes in the wireless approach are placed on top of the brain, it is crucial that they are firmly fixed to the skull. Due to the weight of the recording system, electrodes may detach, which cannot be repaired without removing the whole electrode/dental cement assembly. Therefore, make sure to create drill holes slightly smaller than the diameter of the recording electrodes, and air-dry the skull carefully before fixing the electrodes with dental cement.
7. Since we use a multi-channel recording approach, it is critical to always connect the electrodes in the same order, and to assign each electrode to the same channel throughout the entire procedure. In this way, the recordings from each channel will always be allocated to the same electrode, facilitating inter-experiment data comparison.
8. Electrostatic noise might interfere with the EEG recordings, depending on the setup and its position in the room (Figures 3A,B). Using the wireless approach, electrostatic noise is not a major concern, as all data is collected in a closed system within the recorder. The wireless recorder is built in such a way that all electrodes are grounded, when placed properly. To avoid electrostatic noise, the individual electrodes must not touch each other. For the wired EEG approach electrostatic noise may be a bigger concern, though this fully depends on the surroundings in which the animals are recorded. If electrostatic noise occurs, a Faraday cage proved to be sufficient to solve the problem in our case (Figure 3C).

## Anticipated results and discussion

### Intrahippocampal administration of KA as an alternative to systemic convulsants

This protocol describes the use of intrahippocampal KA, in multiple doses, to elicit epileptiform activity and SE. The highest dose (50 nL, 20 mM) triggers SE similar to systemic KA administration models, however the origin of the seizures, and their behavioral manifestations are different. Locally delivered KA may act on KA and AMPA receptors located both pre- and post-synaptically in pyramidal and granule cells, regulating neurotransmission (Vincent and Mulle, 2009), In contrast, systemic convulsants may act on the whole brain and target different circuits in brain regions enriched in KA receptors, including the cerebral cortex or the amygdala (Vincent and Mulle, 2009). In our intrahippocampal administration model, we have observed largely the same effects (seizure severity, epileptiform activity monitored by EEG, gliosis, effects on the hippocampal neural stem cells) independently of small differences in the coordinates used to inject KA into the DG, supporting the idea that a common circuit is activated. As a result, the high dose of KA is an excellent model of mTLE, as it resembles some of its pathological hallmarks, such as changes in gene expression, induction of reactive gliosis, granule cell dispersion, apoptosis, inflammation, the occurrence of chronic recurrent seizures, and deregulation of the neurogenic process (Schouten et al., 2015; Sierra et al., 2015).

Systemic delivery usually requires multiple injections of the chemoconvulsant until the point in behavioral seizures quantified by the Racine scale can be observed (McLin and Steward, 2006). Thereby, the number of injections needed varies largely per animal, making the KA dose a factor virtually impossible to standardize under these circumstances and resulting in experimental variability that may be associated with result irreproducibility (Hellier et al., 1998; McLin and Steward, 2006). Using the protocol described herein, the dose of KA administered locally can be tightly controlled, allowing the characterization of dose and seizure intensity-dependent effects.

### Low-grade epileptiform activity induction

Whereas other classical models strive to elicit full-blown SE and subsequent chronic recurrent seizures, our model also provides the possibility to induce lower grade epileptiform activity, depending on the KA dose locally delivered, which has its own effects on the hippocampal niche (Sierra et al., 2015). Given the large inter-animal susceptibility when KA is administered systemically, it is virtually impossible to accurately standardize non-SE inducing KA doses, which may generate unwanted experimental variability. The intrahippocampal KA administration described here offers the opportunity to treat multiple animals with the same convulsive or non-convulsive dose of KA, as the local KA dosage can be tightly regulated, allowing the detailed study of the effect of non-seizure-inducing KA-induced neuronal hyperactivity in the hippocampus and the effects of varying seizure intensities as well (Schouten et al., 2015; Sierra et al., 2015).

### Between and within strain susceptibility

Though not new, the intrahippocampal KA model has not been extensively used in mice. However, multiple studies have already made use of this model in rats, where it has been validated as a good epilepsy model on multiple levels, such as SE induction, latency, rise of recurrent chronic seizures, and pathological hallmarks (Cavalheiro et al., 1982, 1983; French et al., 1982; Cook and Crutcher, 1986; Davenport et al., 1990; Leite et al., 1996; Rattka et al., 2013). Keeping in mind the strain-specific KA vulnerability and within-strain individual differences in KA vulnerability in systemically treated mice, systemic administration of KA presents several limitations as epilepsy model in mice. Unfortunately, because of its limited use, no data is yet available on strain differences in KA vulnerability using intrahippocampal injections, as we describe here. Thus, far, only mice on a C57Bl6 background have been used in an experimental setup (Schouten et al., 2015; Sierra et al., 2015). However, our previous observations suggest that the within-strain variability is significantly reduced using local administration protocols (Schouten et al., 2015; Sierra et al., 2015). Together with the numerous possibilities that transgenic mouse lines offer, the use of intrahippocampal KA provides new opportunities for the preclinical epilepsy field. We propose that the intrahippocampal KA model should be considered a suitable and easy to standardize epilepsy model used for mice, which will improve inter-center reproducibility and provide a complementing method for the systemic application of KA.

### Local damage

One factor to consider when using the intrahippocampal KA model is the surgical procedure needed to induce SE. This is especially the case for experiments in which no recording electrodes are placed, since these would not need any surgical procedure when KA would be administered systemically.

Though only a short procedure, anesthetics may influence the effects of KA and there is always a risk of damage to the brain. Therefore, it is important to carefully select the anesthetic of choice, and optimize the surgery protocol. In our hands, no clear local damage can be found in the hippocampus besides the presence of the needle tract due to the stereotaxic surgery procedure. We have not found any indications of inflammatory markers surrounding the needle tract, confirming that the sterile invasive procedure may not result in significant local inflammatory response, which could be a confounding factor in epilepsy models (Sierra et al., 2015).

In conclusion, the protocol for intrahippocampal administration of KA described here offers the advantage of allowing a controlled release of the convulsant dose of choice, in the specific location of interest, and in a variety of transgenic mouse models, while limiting the local damage induced in the injection site. Nonetheless, it should be noted that this model has not been as thoroughly characterized as the systemic models, in particular regarding the latent and chronic periods (duration and frequency of seizures, etc), and thus further characterization is needed in this respect. Overall, the intrahippocampal administration of KA offers a great potential to understand the underlying pathophysiology of epilepsy and mTLE.

## Author contributions

AS, JE, AA, and MM designed the intrahippocampal KA protocol and the wired EEG recording setup. PB and CF optimized the intrahippocampal KA protocol and designed the wireless EEG recording setup. PB, AS, and CF wrote the manuscript.

## Funding

This work was supported in part by the grant H64.09.016 from the Innovational Research Incentives Scheme VIDI, The Netherlands Organization for Scientific Research (NWO) to CF. This work was supported by grants from the Spanish Ministry of Economy and Competitiveness with FEDER funds to AS (BFU2015-66689, RYC-2013-12817).

### Conflict of interest statement

The authors declare that the research was conducted in the absence of any commercial or financial relationships that could be construed as a potential conflict of interest.

## Footnotes

^#^Kainic acid is a potent neurotoxin and thus has to be handled with care and using proper personal protective equipment such as standard disposable latex laboratory gloves, facial mask, and biosecurity goggles. Dispose any residual solutions according to local regulations.

^*^Braided silk sutures may induce an inflammatory response in the skin, thereby increasing animal discomfort. Therefore, the use of PVDF sutures is advised (Kakoei et al., 2010).
